# Predicting saturated and near-saturated hydraulic conductivity using artificial neural networks and multiple linear regression in calcareous soils

**DOI:** 10.1371/journal.pone.0296933

**Published:** 2024-01-10

**Authors:** Hasan Mozaffari, Ali Akbar Moosavi, Mohammad Amin Nematollahi

**Affiliations:** 1 Department of Soil Science, College of Agriculture, Shiraz University, Shiraz, Iran; 2 Department of Biosystems Engineering, College of Agriculture, Shiraz University, Shiraz, Iran; UNITEN: Universiti Tenaga Nasional, MALAYSIA

## Abstract

Hydraulic conductivity (*K*_*ψ*_) is one of the most important soil properties that influences water and chemical movement within the soil and is a vital factor in various management practices, like drainage, irrigation, erosion control, and flood protection. Therefore, it is an essential component in soil monitoring and managerial practices. The importance of *K*_*ψ*_ in soil-water relationship, difficulties for its measurement in the field, and its high variability led us to evaluate the potential of stepwise multiple linear regression (SMLR), and multilayer perceptron (MLPNNs) and radial-basis function (RBFNNs) neural networks approaches to predict *K*_*ψ*_ at tensions of 15, 10, 5, and 0 cm (*K*_15_, *K*_10_, *K*_5_, and *K*_0_, respectively) using easily measurable attributes in calcareous soils. A total of 102 intact (by stainless steel rings) and composite (using spade from 0–20 cm depth) soil samples were collected from different land uses of Fars Province, Iran. The common physico-chemical attributes were determined by the common standard laboratory approaches. Additionally, the mentioned hydraulic attributes were measured using a tension-disc infiltrometer (with a 10 cm radius) in situ. Results revealed that the most of studied soil structure-related parameters (soil organic matter, soluble sodium, sodium adsorption ratio, mean weight diameter of aggregates, pH, and bulk density) are more correlated with *K*_5_ and *K*_0_ than particle-size distribution-related parameters (sand, silt, and standard deviation and geometric mean diameter of particles size). For *K*_15_ and *K*_10_, the opposite results were obtained. The applied approaches predicted *K*_15_, *K*_10_, *K*_5_, and *K*_0_ with determination coefficient of validation data (R^2^_val_) of 0.52 to 0.63 for SMLR; 0.71 to 0.82 for MLPNNs; and 0.58 to 0.78 for RBFNNs. In general, the capability of the applied methods for predicting *K*_*ψ*_ at all the applied tensions was ranked as MLPNNs > RBFNNs > SMLR. Although the SMLR method provided easy to use pedotransfer functions for predicting *K*_*ψ*_ in calcareous soils, the present study suggests using the MLPNNs approach due to its high capability for generating accurate predictions.

## Introduction

Increased population pressure and the necessity of efficient water management have recently led attentions to reuse arid and semiarid soils as potential productive soil resources [1]. Hydraulic conductivity (*K*_*ψ*_) is an important attribute in designing and monitoring drainage and irrigation systems and to characterize many aspects of water flow within unsaturated soils such as irrigation, aquifer recharge, infiltration of rainfall and runoff, nutrient transportation, pollutants, and pesticides, and water balance [2–4]. There are some crucial factors such as pores size distribution and soil structure [5,6], bulk (apparent) density (BD) [7], particle-size distribution (PSD) [8], and soil organic matter, (SOM) content [9] that can significantly affect *K*_*ψ*_.

The values of *K*_*ψ*_ are highly depending on the methods used for their determination [1]. Moreover, its measurement both in the field (by double rings, tension-disk infiltrometer, or Guelph permeameter) and laboratory (using the constant and falling head approaches) is a laborious, challenging, and costly process [10]. Consequently, various efforts have been made to indirectly determine saturated and, to a lesser extent, unsaturated or near-saturated hydraulic conductivity. This has been achieved through creation of pedotransfer functions (PTFs), which used easily measurable soil attributes. These easily measurable soil attributes include soil texture and PSD parameters, BD, mean weight diameter of aggregates (MWD), SOM content, calcium carbonate equivalent (CCE), and others. To estimate saturated and unsaturated hydraulic conductivity, different approaches have been used in literature like, linear [11–14] and non-linear [15,16] regressions, and different machine learning algorithms [13,16–19]. Nevertheless, the potential of PTFs to accurately predict *K*_*ψ*_ is limited due to not incorporating soil structure, which is a crucial factor influencing hydraulic conductivity [20].

Artificial neural networks (ANNs), ANNs-based PTFs, and other related approaches have been widely employed as powerful modeling tools in various fields of science and engineering [21–23]. The ANNs are inspired by the human brain which is able to simulate the complex relation between input and output data [24]. According to literature, ANNs typically exhibit higher efficiency and potentials to estimate hardly measurable soil attributes rather than conventional regression approaches [25–27]. There are some advantages of ANNs approaches compared to traditional PTFs. For instance, in the ANNs approaches there is: i) no need to pre-assumptions for modeling; ii) no need to priori assumptions of data distribution; iii) high capability to model complex and non-linear behaviors; and iv) high compatibility with missing and noisy data [24]. However, ANNs approaches in comparison to conventional PTFs have some weaknesses like: i) “black boxes” modeling; ii) necessity for a large number of data to obtain the optimal weights and biases of the network; and iii) necessity for trial and error approaches to select the most suitable parameters in their structures [28,29].

Two feed-forward NNs (FFNNs), including multilayer perceptron (MLPNNs) and radial-basis function (RBFNNs), have been commonly used among various types of ANNs as efficient tools for recognizing patterns or approximate functions [30,31].

Although ANNs have been used to predict saturated hydraulic conductivity (*K*_0_) in several studies [32–37], there is a limitation in literature which applied ANNs to predict unsaturated hydraulic conductivity especially in calcareous soils. In this regard, Moosavi and Sepaskhah [38] in the Fars Province of Iran, found that the most accurate prediction of *K*_*ψ*_ at tensions of 20, 15, 10, 6, 3, and 0 cm were obtained with a four-layer MLPNNs with three nodes in the first and four nodes in the second hidden layers. Sihag [39] in sandy soils of India reported that the prediction of unsaturated hydraulic conductivity by back propagation algorithm based on ANNs approach works better than fuzzy logic. Jian et al. [40] in the USA soils found the two-hidden layers MLPNNs approach with the first and the second layers of five and three nodes had a good performance to predict *K*_*ψ*_ at 2 cm tension when trained with soil texture components (i.e., clay, silt, and sand percentages).

In addition to the mentioned limitation, there are no reports about the prediction of field measured near-saturated *K*_*ψ*_ using multiple linear regression (MLR), MLPNNs, and RBFNNs approaches and comparing their modeling potential. Therefore, novel aspects of the present study were covering the potential of two common FFNNs to predict saturated and near-saturated *K*_*ψ*_ in calcareous soils and also comparing their capability with that of the MLR approach. In general, this study aimed to: i) predict *K*_*ψ*_ of calcareous soils at tensions of 15, 10, 5, and 0 cm using MLPNNs and RBFNNs along with MLR, and ii) compare capability of the mentioned approaches and their accuracies to predict *K*_*ψ*_ in calcareous soils.

## Materials and methods

### Study area

Totally 102 locations were randomly selected in the Fars Province, Iran to measure physico-chemical and hydraulic attributes of the soils, which cover the most relevant land uses and soil types. It should be noted that for developing reliable models to predict specific soils attribute, the number of input data, which is largely depends on difficulties and costs for its determining, is very important. For this purpose, normally the 100 data are trustworthy and appropriate in soil science studies. Of course, higher numbers of input data result in more reliable models. Still, due to difficulties and spending lots of time for measuring the *K*_*ψ*_, the 102 data would be appropriate for performing the present study. In addition, the random selection of experimental points while considering different land uses can help to include almost all soil types with different hydraulic, physical, and chemical attributes in the study. Therefore, the obtained results can be used in larger areas with similar soil conditions.

General descriptions of the study region (Fars Province) and the soils were summed up in Table 1. The Fars Province, in south and southwest regions of Iran, has a great potential to grow many types of agricultural plants like wheat, barley, corn, rice, alfalfa, etc. On the other hand, there are different types of land uses, like croplands, pasture, garden, woodland, etc. in the mentioned Province. The soils of Fars Province are calcareous and relatively calcareous (with a CCE contents of 12.5–70.6% based on our data in the present study), which can be a good representative for calcareous soils in the Middle East. Due to these mentioned reasons, the Fars Province was selected as target area for performing the present study.

**Table 1:** General descriptions of the study region and the studied soils.

Characteristic	Description	References
Location	Fars Province	-
Geographical coordinates	50° 30′ to 55° 38′ E and 27° 03′ to 31° 42′ N	-
General climate	Arid and semi-arid	-
Climate regime based on Köppen-Geiger	BWh, BSk, BSh	[41]
Mean annual temperature	17.5°C	[42]
Annual precipitation	5 to 100 cm	[43]
Elevation from the mean sea level	0.5 to 4 km	[44]
Soil moisture regimes	Xeric, ustic, aridic	[45]
Soil temperature regimes	Mesic, thermic, hyperthermic	[45]
Parent material	Soluble dolomite and calcite limestone	[46]
Studied soil types according to Taxonomy classification	Inceptisols (71 soils), Aridisols (18 soils), and Entisols (13 soils)	[44,47]
Studied land uses	Croplands (wheat, barley, corn, rice, and fallow), pasture, woodland (oak forests)	-

At each of the 102 selected locations, water infiltration was measured using a tension-disk infiltrometer apparatus. Intact soil samples of 3.5 cm diameter and 2 cm height (using stainless steel rings) and 1 kg composite samples (using spade) were taken from the depth of 0 to 20 cm to measure soil physico-chemical attributes.

### Analyzing selected physico-chemical attributes of the soils

Air-dried soil samples, that were ground and sieved to sizes of 8 and 2 mm, were prepared to determine the selected physico-chemical attributes using standard laboratory procedures. Generally, in the present study the selected physico-chemical attributes were divided in two groups: i) PSD-related parameters, and ii) structure-related parameters. The PSD-related parameters contain sand, silt, clay, geometric mean (*d*_*g*_) and geometric standard deviation (*σ*_*g*_) of particles size diameter, and fractal dimension (D). The PSD and its related parameters affect pores size distribution due to the sizes of primary particles. For instance, water infiltrates faster in the coarse-textured soils compared with the fine-textured soils due to having a high content of sand. On the other hand, existing clay content in soil is very essential for aggregation. Therefore, the PSD-related attributes may have significant effects on hydraulic parameters, like *K*_*ψ*_. Subsequently, the structure-related parameters contain BD, MWD, pH, SOM, CCE, electrical conductivity (EC), water-soluble sodium (Na^+^), potassium (K^+^), calcium (Ca^2+^), and magnesium (Mg^2+^), and sodium adsorption ratio (SAR). In general, the mentioned structure-related parameters may affect the pores size distribution of soils, due to their effects on aggregation and disaggregation. The *K*_*ψ*_ values may therefore be affected by these structure-related parameters.

The soil PSD and texture were determined using the combined sedimentation (hydrometer) and wet-sieving methods [48]. More specifically, the PSD of the soils was obtained based on measuring the density of soil-water suspension at 120, 300, and 600 sec, and at 1, 3, 6, and 24 h by hydrometer (for particles of less than 0.05 mm diameter) and remaining particles on sieves with opening diameters of 1, 0.5, 0.15, and 0.05 mm (for sand fraction) [48].

The BD of soils was measured using stainless steel rings with 3.5 cm diameter and 2 cm height [49]; MWD using a series of 8 sieves with opening diameters of 4, 2, 1, 0.8, 0.6, 0.4, 0.2, and 0.075 mm by dry-sieving method [50]; pH of saturated paste using pH-meter (glass electrode) [51]; SOM content using oxidation of soil organic carbon by potassium dichromate and titration with ammonium ferro sulfate as reductant (Walkley-Black wet oxidation) method [52]; CCE using back titration with hydrochloric acid (HCl) method [53]; EC of saturated extract using conductometer (EC-meter) [54]; water-soluble Na^+^ and K^+^ of saturated extract using a flame photometer [55]; and water-soluble Ca^2+^ and Mg^2+^ of saturated extract using titration with EDTA method [56].

In addition, the PSD data was used to calculate some indices like, *d*_*g*_ and *σ*_*g*_ [57], and D [58]. Furthermore, SAR was calculated using the values of water-soluble Na^+^, Ca^2+^, and Mg^2+^ (for details see Mozaffari et al., [59]).

### Infiltration experiments

**Fig 1:**
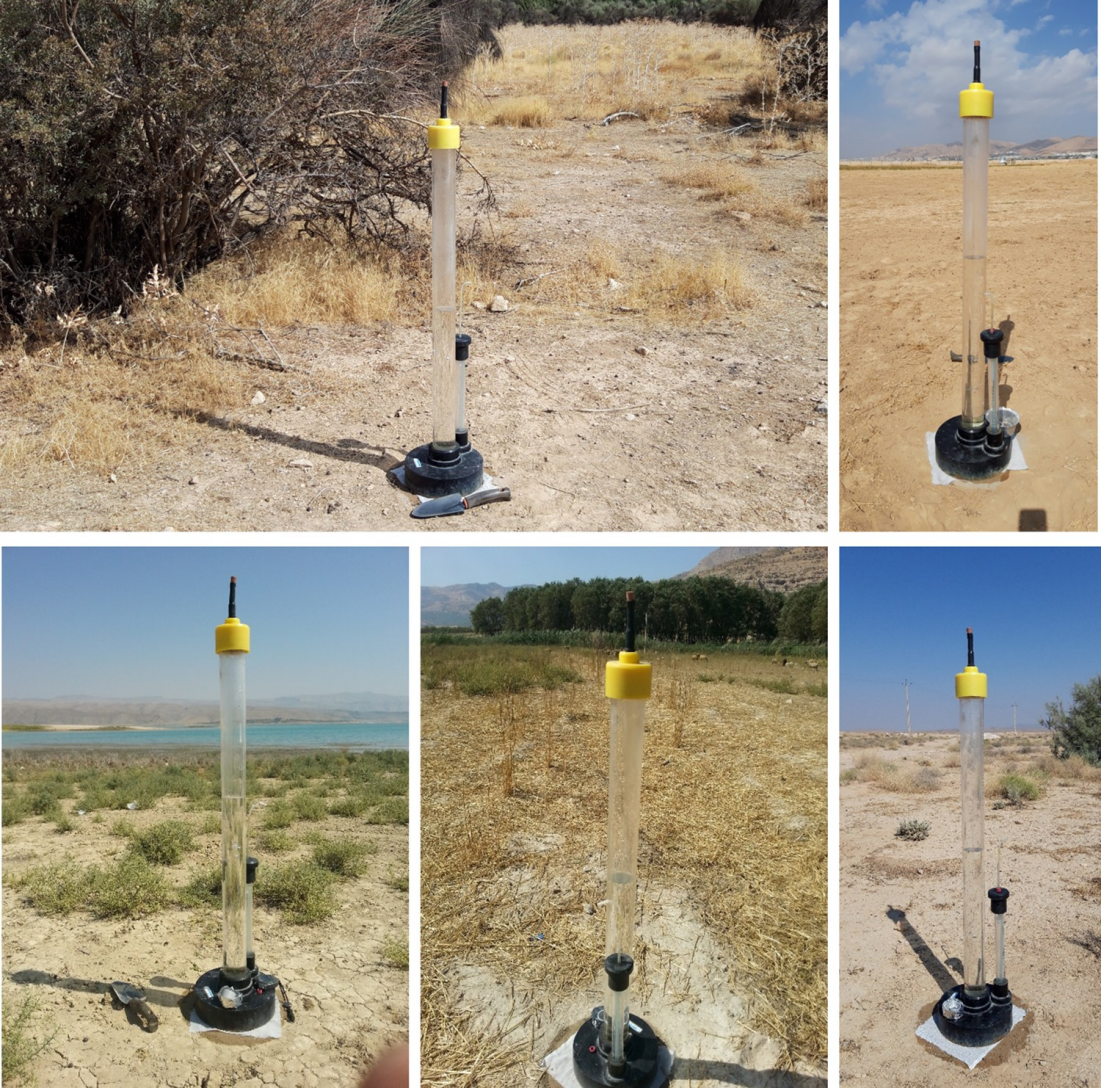
Measurement of *K*_*ψ*_ by tension-disk infiltrometer at different land uses.

A single-disc tension infiltrometer with 10 cm radius was used to conduct the infiltration experiments (Fig 1). At first, at each experimental location, the residues of the plant materials were gently eliminated from the soil surface without causing any alteration to the soil structure. To ensure a suitable hydraulic connection between the soil surface and the disc membrane of the infiltrometer, we used a thin contact layer (nearly 0.5 cm) of fine sand with particle diameters of 0.1 to 0.25 mm. Fresh water (with EC of 0.6 dS m^-1^ and SAR of 0.5 meq^0.5^ L^-0.5^) was used to fill the device reservoir for conducting the infiltration experiments. After that, the infiltrometer was gently put onto the layer of sand and fixed. The experiment was started with the intended maximum tension, which was considered as 15 cm during this study. The Mariotte tubes and air tower were used for setting the applied tensions. In the present study, for performing infiltration test, four successive tensions including 15, 10, 5, and 0 cm were applied at each experimental site. At each tension, the height of infiltrated water was manually recorded at the time intervals of 0.25 min (for the first 5 min) and 1 min to reach the steady-state conditions. Consider that, the steady-state conditions are achieved when at least five successive infiltration rates became similar [60]. The time needed to reach the steady state conditions varies for different soils, and usually falls between 20 to 60 min at each applied tension.

### Calculating near-saturated and saturated hydraulic conductivity (*K*_*ψ*_)

The *K*_*ψ*_ at different applied tensions (*ψ*) was calculated using the method introduced by Ankeny et al. [61]. According to their theory, the *K*_*ψ*_ was determined based on the collected infiltration data using well-known Wooding’s approach [62]. In this particular approach, it is assumed that the variation of *K*_*ψ*_ with respect to *ψ* (as described in Eq 1) follows an exponential pattern. This assumption holds true when water enters the soil from a circular source with a fixed radius "r" under a constant tension [62–64]:

$$K_{\psi }=K_{0}\;\exp(\alpha \;\psi )$$ (1)

where *K*_0_ and *α* are hydraulic conductivity (L T^-1^) at zero cm *ψ* and the sorptive number (L^–1^), respectively [65,66]. Wooding [62] derived the subsequent analytical solution:

$$Q_{\psi }=\pi \;r^{2}\;K_{\psi }+\frac{4\;\pi \;r^{2}\;K_{\psi }}{\pi \;r\;\alpha }=\pi \;r^{2}\;K_{\psi }(1+\frac{4}{\pi \;r\;\alpha })$$ (2)

where *Q*_*ψ*_ represents the volume (L^3^) of water infiltrates into the soil per unit time (T), at tension *ψ* under steady-state conditions and *r* denotes radius (L) of the disc infiltrometer apparatus.

Based on Ankeny et al. [61] analysis, by applying Wooding’s solution for unsaturated steady-state conditions and substituting *K*_*ψ*_ with Eq (1), as well as replacing *ψ*_*i*_ and *ψ*_*i‏*+1_ into the resulting equation, we would obtain Eqs (3) and (4).


$$Q_{\psi_{i}}=\pi \;r^{2}\;K_{0}\;\exp(\alpha \;\psi_{i})+\frac{4\;\pi \;r^{2}\;K_{0}\exp(\alpha \;\psi_{i})}{\pi \;r\;\alpha }$$ (3)

$$Q_{\psi_{i+1}}=\pi \;r^{2}\;K_{0}\exp(\alpha \;\psi_{i+1})+\frac{4\;\pi \;r^{2}\;K_{0}\exp(\alpha \;\psi_{i+1})}{\pi \;r\;\alpha }$$ (4)

Ankeny et al. [61] solved Eqs (3) and (4) simultaneously as follows:

$$K_{\psi_{i}(1)}=\frac{Q_{\psi_{i}}}{\pi \;r^{2}+2\;\Delta \psi \;r(\frac{Q_{\psi }{}_{{}_{i}}+Q_{\psi_{i+1}}}{Q_{\psi }{}_{{}_{i}}})/(\frac{Q_{\psi }{}_{{}_{i}}-Q_{\psi_{i+1}}}{Q_{\psi }{}_{{}_{i}}})}$$ (5)

$$K_{\psi_{i}(2)}=\frac{Q_{\psi }{}_{{}_{i+1}}K_{\psi_{i}(1)}}{Q_{\psi }{}_{{}_{i}}}$$ (6)

In present study, the values of 0 and 5, 5 and 10, and 10 and 15 cm tensions were used to solve three equations simultaneously. Ankeny et al. [61] stated that the arithmetic average value has the best compatibility with the *K*_*ψ*_, where $K_{\psi_{i}}$ is obtained from the (*ψ*_*i*_, *ψ*_*i*+1_) rate pair ($K_{\psi_{i}}=\frac{K_{\psi }{}_{{}_{i+1}}+K_{\psi_{i}}}{2}$). Eqs (7) to (10) indicate how *K*_0_, *K*_5_, *K*_10,_ and *K*_15_ were practically calculated [61]:

$$K_{0}=K_{0}{}_{\left(0,5\right)}$$ (7)

$$K_{5}=\frac{K_{5}{}_{\left(0,5\right)}+K_{5}{}_{\left(5,10\right)}}{2}$$ (8)

$$K_{10}=\frac{K_{10(5,10)}+K_{10(10,15)}}{2}$$ (9)

$$K_{15}=K_{15}{}_{\left(10,15\right)}$$ (10)

### Stepwise multiple linear regression (SMLR)

The SMLR, which still is one of the most commonly used and standardized approaches for developing PTFs [20], was employed for predicting *K*_*ψ*_ as described below:

$$K_{\psi }=a_{0}+a_{1}\;V_{1}+a_{2}V_{2}+\ldots +a_{N}\;V_{N}$$ (11)

where *V*_1_ to *V*_N_ are the independent variables (easily measurable soil attributes), *a*_0_ to *a*_*N*_ denote regression coefficients, and *N* shows the number of independent variables. The significant predictor variables were selected using a forward approach. For developing SMLR-PTFs, the *K*_*ψ*_ at different applied tensions and easily measurable soil attributes were selected as dependent and independent variables, respectively. The STATISTICA (version 8.0) software package was used for developing SMLR-PTFs.

### Artificial neural networks (ANNs)

#### Multilayer perceptron NNs (MLPNNs)

The ANNs are a subset of machine learning, which consist of several layers and processing elements called neurons. Two unknown parameters including weights (*w*_*ji*_) and biases (*b*_*j*_) in the structure of the MLPNNs should be adjusted by the training process [24,67]. For this purpose, an error function, which is a function of weights and biases, should be minimized as follows:

$$E=\sum_{i=1}^{m}\sum_{j=1}^{n}{\left(P_{ij}-O_{ij}\right)}^{2}$$ (12)

where *O*_*ij*_ and *P*_*ij*_ denote the observed (measured) and predicted values of dependent variable, respectively. *m* and *n* are the number of output and data fed into the networks, respectively. An iterative back-propagation algorithm is usually used to adjust the *w*_*ji*_ as following:

$$w_{ji}(k+1)=w_{ji}(k)+\Delta w_{ji}(k)$$ (13)

A generalized delta-learning rule is applied to determine the Δ*w*_*ji*_ (*k*) values [24,67].

In the current study, for training MLPNNs, various hidden layers of different numbers of nodes (neurons) were examined. The MATLAB software package was used to select the most effective hidden layers combination and their number of neurons, and to predict the *K*_*ψ*_. For training the MLPNNs, an architecture with two hidden layers were selected for all *K*_*ψ*_ that includes 8 and 12, 6 and 11, 7 and 10, and 9 and 14 neurons in the first and second layers to estimate the *K*_0_, *K*_5_, *K*_10_, and *K*_15_, respectively.

Different transfer functions and learning (training) algorithms were tested in the mentioned MLPNNs to achieve the lowest value of Eq (12). The Levenberg-Marquardt algorithm was employed as learning (training) algorithm in the structure of the used MLPNNs for estimating all *K*_*ψ*_. In addition, the sigmoid transfer function was used in the hidden layer, whereas linear transfer function was employed for the output (target) layer.

#### Radial-basis function NNs (RBFNNs)

The RBFNNs is another type of FFNNs that composed of two layers with simple structure and fast in learning. There are two main stages for learning process in RBFNNs: i) obtaining the centers of the clustered input variables (data) by the unsupervised approaches [24,67,68], and ii) determining the weights of the network using the least square approach. The RBFNNs output is computed as:

$$Y_{q}(X)=\sum_{K_{\psi }=1}^{\varepsilon }w_{qj}\;\varphi_{j}(\|X-U_{j}\|)+b_{q}$$ (14)

where *X* and *U*_*j*_ show the input vector and the vector corresponding to the center of the RBF *φ*_*j*_ (as transfer function), respectively. The Gaussian function is commonly employed as a basis function for the RBFNNs, which is given as:

$$\varphi_{j}(\left\|X-U_{j}\right\|)=\exp(-\frac{\left|X-U_{j}\right|}{2\;\sigma^{2}})$$ (15)

where *σ* denote the spread of the RBF.

### Data analysis

At first, the selected hydraulic, physical, and chemical attributes were subjected to calculate the descriptive statistics and normality test using Excel (version 2019) and SPSS (version 26) software packages. Attributes that did not follow normal distribution were transformed using the natural logarithm (ln) transformation to be closer to the normal distribution. To evaluate the potential of selected modeling approaches to predict *K*_*ψ*_ at different applied tensions by easily measurable soil attributes, the total of 102 laboratory/field measured soil attributes were randomly divided to 75% and 25% of the dataset as the calibration and validation subsets, respectively [38,69–71]. Furthermore, in order to ensure that: i) both validation and calibration subsets of *K*_*ψ*_ have approximately the same distributions, and ii) the mean values of the aforementioned subsets have no significant difference; the 1:1 line (using Excel software) and *t-*test analysis (using STATISTICA software) were used, respectively [72]. After that, the *K*_*ψ*_ values at different applied tensions (as dependent variables) and studied physico-chemical attributes (as independent variables) related to calibration subset were imported to the STATISTICA (version 8) software package for developing PTFs using forward-SMLR approach. The most significant and effective easily measurable attributes were appeared in four developed SMLR-PTFs to predict *K*_*ψ*_ at different applied tensions. To avoid the complexity of the NNs models, the appeared physico-chemical attributes in developed SMLR-PTFs were imported to MATLAB software package as independent variables for predicting *K*_*ψ*_ using MLPNNs and RBFNNs models. It is worth mentioning that, all applied models were developed by the calibration subset and then tested by the validation subset.

### Statistical evaluation of the models

Several statistical indices including the determination coefficient (R^2^), the Nash-Sutcliffe coefficient (NS), the residual prediction deviation (RPD), and the normalized root mean square error (NRMSE) were employed to assess and compare the performance of the models (Eqs 16 to 19):

$$R^{2}=\frac{{\left(\sum_{i=1}^{n}\left(O_{i}-\bar{O})(P_{i}-\bar{P}\right)\right)}^{2}}{\sum_{i=1}^{n}{\left(O_{i}-\bar{O}\right)}^{2}\sum_{i=1}^{n}{\left(P_{i}-\bar{P}\right)}^{2}}$$ (16)

$$\mathrm{NS}=1‐\frac{\sum_{i=1}^{n}{\left(O_{i}‐P_{i}\right)}^{2}}{\sum_{i=1}^{n}{\left(O_{i}‐\bar{O}\right)}^{2}}$$ (17)

$$\mathrm{RPD}=\frac{Sd}{SEP}$$ (18)

$$\mathrm{NRMSE}(\%)=\frac{\sqrt{\sum_{i=1}^{n}\frac{{\left(O_{i}-P_{i}\right)}^{2}}{n}}}{\bar{O}}\times 100$$ (19)

where *P*_*i*_ and *O*_*i*_ show the predicted and measured (observed) data, *n* is number of data, Sd and SEP denote the standard deviation and standard error of the measured data (SEP is equal to RMSE of the predicted data). Table 2 shows the reported classifications for different ranges of NRMSE, NS, and RPD performance criteria. The statistical calculations were performed using the MATLAB software package.

**Table 2:** Different classifications for the selected performance criteria.

NRMSE^†^		NS^††^		RPD^†††^	
Range	Classification	Range	Classification	Range	Classification
0–10%	Excellent	0.90–1	Very good	≥ 2.5	Excellent
10–20%	Good	0.80–0.90	Good	2.0–2.5	Very good
20–30%	Fair	0.65–0.80	Acceptable	1.8–2.0	Good
> 30%	Poor	< 0.65	Unsatisfactory	1.4–1.8	Fair
-	-	-	-	1.0-.1.4	Poor
-	-	-	-	< 1	Very poor

†: Normalized root mean square error (After Bannayan and Hoogenboom [73]).

††: The Nash-Sutcliffe coefficient (After Ritter and Muñoz-Carpena [74]).

†††: The residual prediction deviation (After Mouazen et al. [75]).

## Results and discussion

### Summary statistics

**Fig 2:**
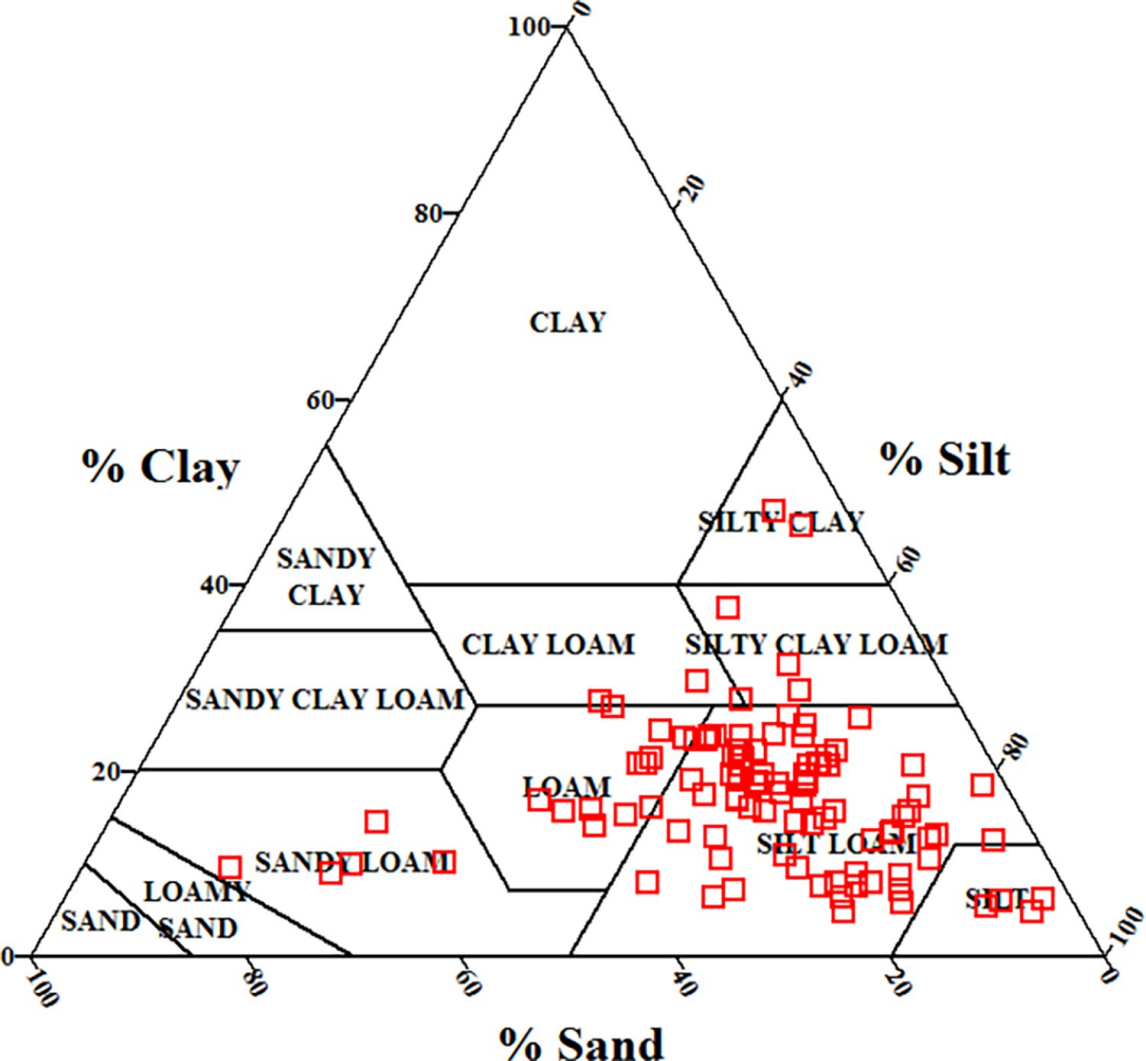
The United State Department of Agriculture (USDA) soil textural classes (*n* = 102).

According to Fig 2, the soils included a broad range of textures (mainly silt loam and loam classes). Therefore, it can be expected that the PSD-related parameters (clay, silt, sand, *d*_*g*_, *σ*_*g*_, and D) be significant and effective parameters for predicting *K*_*ψ*_. It should be noted that we used the United State Department of Agriculture (USDA) guideline to classify the soil texture components, which means that a particle with diameter of less than 0.002 mm is clay; with diameter of 0.002 to 0.05 mm is silt; and with diameter of 0.05 to 2 mm is sand [76]. The *d*_*g*_, *σ*_*g*_, and D are criteria of PSD data. They represent unique features of PSD. For example, with increasing clay content and decreasing sand content, the D values increase and the *d*_*g*_ values decrease [57,77], but their relationships with soil texture components (sand, silt, and clay contents) are not linear. While, the *σ*_*g*_ parameter depends on combination of all texture components in the soil [57]. The primary particles (sand, silt, and clay) contents affect pores size distribution due to their sizes and also their impacts on aggregation. Furthermore, macropores and mesopores play crucial roles in water flow through the soil, especially when it comes to processes such as infiltration and the rapid movement of water, solutes, and pollutants through the soil [78,79]. Therefore, the PSD-related parameters may affect infiltration rate and subsequently *K*_*ψ*_ at different tensions.

The obtained results demonstrated, among studied soil attributes, the D and SAR with coefficient of variations (CV) values of 2.1% and 277%, respectively, represent the lowest and the highest CVs (Table 3). According to classification proposed by Wilding [80], the D, BD, and pH belong to the class of low variability (0% < CV ≤ 15%); *K*_5_, MWD, silt, *σ*_*g*_, and CCE belong to the class of moderate variability (15% < CV ≤ 35%); and the remaining studied soil attributes belong to the class of high variability (CV > 35%). The higher CV values show the greater level of the data dispersion around the mean. Therefore, the high variability of an attribute in a dataset enables the researcher to model that parameter with higher certainty and the resulting model can be used on a larger scale. In contrast, a lower CV value of input data may result in a good model, while the applicability of the model is limited to a small range of target attribute. Of course, it should be noted that some attributes (for instance D, BD, and pH) inherently have low CVs due to the low values of standard deviation in comparison with their mean values. More specifically, the D value normally changes between 2.4 to 3 in soils, which in the present study varied between 2.56 to 2.85. Therefore, the minimum value of CV was obtained for this property. While, in arid and semi-arid regions, like the study area, very high values of EC and water-soluble cations can be observed. In irrigated fields with fresh water, the soluble salts and cations are leached toward the deeper sections of soil profile. While, in the pastures or rainfed fields, the soluble salts and cations of subsoil may migrate to the surface layers of soils through capillary movement and accumulate there. Therefore, the high variability of EC, water-soluble Na^+^, K^+^, Ca^2+^, and Mg^2+^, and SAR may be observed in such arid and semi-arid regions. In this regard, the EC values of 0.26 to 76.7 dS m^-1^ was reported by Mozaffari et al. [76] in the soils of Fars Province, Iran. In addition, moderate and high variabilities of *K*_*ψ*_ in the present study indicate that the obtained models may work well on a larger scale with similar soils.

**Table 3:** The descriptive statistics and related normality test parameters for the selected attributes of the studied soils (*n* = 102).

Property	Unit	Min	Max	Mean	CV (%)	VC	SK	KR	KS^†^
*K*_15_	cm h^-1^	0.084	1.17	0.520	39.5	High	0.604	0.727	0.070 ^ns^
*K*_10_	cm h^-1^	0.097	1.81	0.877	36.1	High	0.350	0.361	0.039 ^ns^
*K*_5_	cm h^-1^	0.790	3.68	2.17	27.4	Moderate	0.091	-0.726	0.075 ^ns^
*K*_0_	cm h^-1^	1.44	10.9	5.72	41.3	High	0.159	-0.966	0.093 ^ns^
MWD	mm	0.374	3.52	1.47	28.6	Moderate	1.10	4.68	0.064 ^ns^
Sand	%	2.28	60.5	23.1	48.1	High	0.897	1.96	0.094 ^ns^
Silt	%	13.6	91.0	59.4	23.7	Moderate	-0.395	0.927	0.061 ^ns^
Clay	%	4.88	47.9	17.5	45.2	High	1.03	2.64	0.079 ^ns^
*d*_*g*_	μm	3.32	32.2	12.6	43.8	High	1.08	1.31	0.130 ^ns^
*σ*_*g*_	mm	2.50	12.2	6.20	27.7	Moderate	0.182	0.616	0.057 ^ns^
D	-	2.56	2.85	2.71	2.10	Low	-0.637	0.326	0.108 ^ns^
BD	g cm^-3^	1.27	1.88	1.56	7.52	Low	0.359	0.619	0.077 ^ns^
pH	-	6.60	7.99	7.47	3.56	Low	-0.836	0.968	0.112 ^ns^
EC	dS m^-1^	0.364	3.18	0.816	58.3	High	2.45	7.28	0.219 *
ln(EC)	-	-1.01	1.16	-0.317	-	-	1.06	0.958	0.129 ^ns^
Na^+^	mg L^-1^	8.28	153	28.7	85.3	High	3.23	13.0	0.202 *
ln(Na^+^)	-	2.11	5.03	3.14	-	-	0.590	0.392	0.061 ^ns^
K^+^	mg L^-1^	1.66	75.2	19.6	92.3	High	1.42	1.12	0.176 *
ln(K^+^)	-	0.507	4.32	2.58	-	-	0.170	-0.859	0.101 ^ns^
Ca^2+^	mg L^-1^	68	720	181	66.5	High	2.58	7.29	0.222 *
ln(Ca^2+^)	-	4.22	6.58	5.06	-	-	1.08	1.18	0.097 ^ns^
Mg^2+^	mg L^-1^	4.80	60.0	12.5	100	High	2.23	4.41	0.225 *
ln(Mg^2+^)	-	1.57	4.09	2.21	-	-	1.08	0.158	0.132 ^ns^
SAR	(meq L^-1^)^0.5^	0.185	34.1	2.01	277	High	4.68	22.88	0.404 *
ln(SAR)	-	-1.69	3.53	-0.401	-	-	1.83	3.46	0.131 ^ns^
CCE	%	12.5	70.6	45.7	25.3	Moderate	-0.365	0.375	0.098 ^ns^
SOM	%	0.170	4.26	1.74	56.8	High	0.733	-0.055	0.104 ^ns^

†: ns demonstrate no significant difference with the normal distribution and * indicates significant difference with the normal distribution at the probability level of 5%.

Furthermore, the Kolmogorov-Smirnov test of normality showed the Na^+^, EC, SAR, K^+^, Mg^2+^, and Ca^2+^ departed from normal distribution (Table 3). Normal distribution of the input data is an essential assumption for applying the MLR modeling procedure. There are different types of data transformations, like taking logarithm or natural logarithm, square rooting, squaring, etc. Therefore, the mentioned chemical attributes were transformed using the natural logarithm (ln or logarithm to the base *e*) function for being closer to the normal distribution.

### Correlation between studied attributes

**Fig 3:**
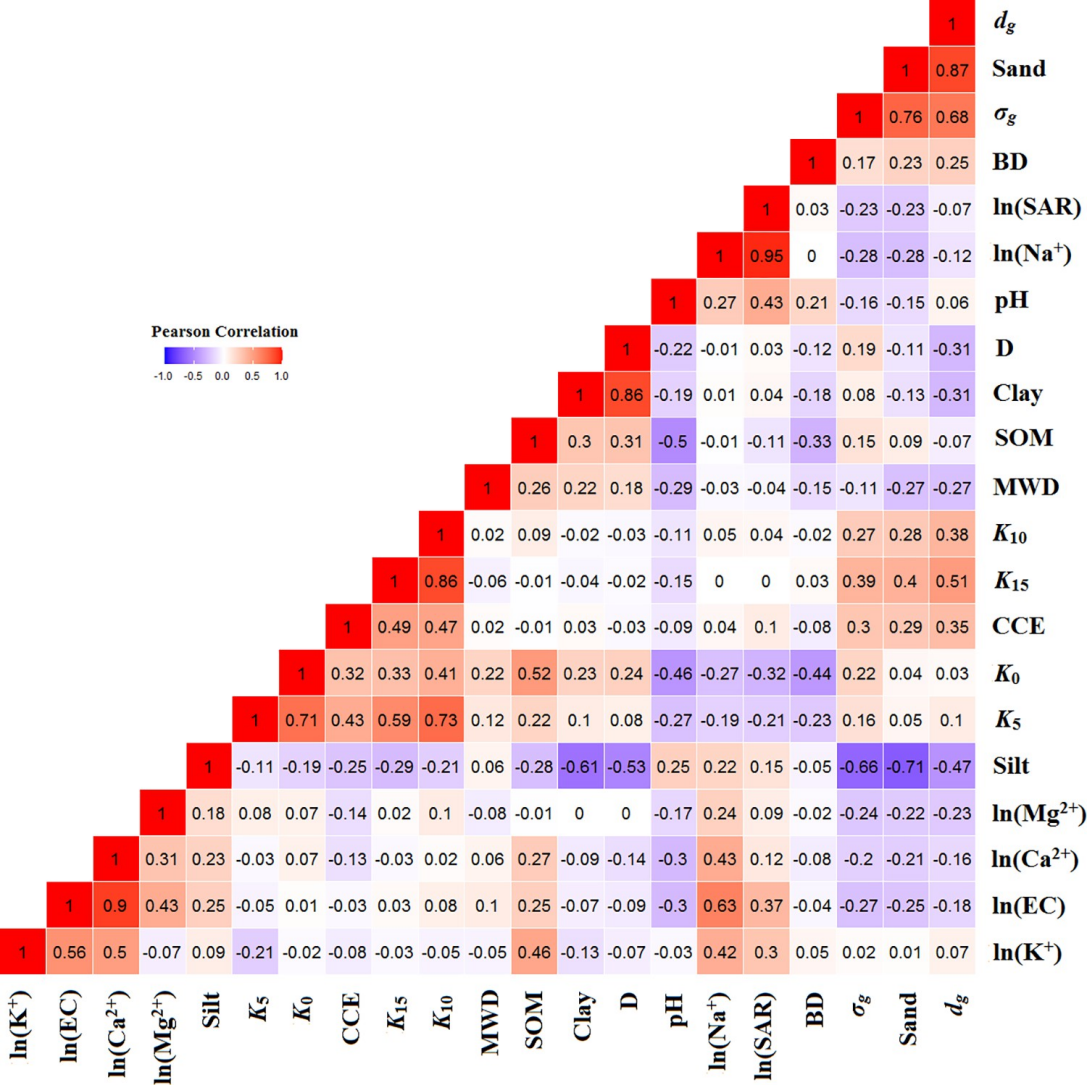
Pearson’s correlation coefficients (R) among studied soil attributes.

The Pearson’s correlation coefficients (R) between the soil attributes are shown in Fig 3. As it was expected, the PSD-related parameters (i.e., silt, sand, *σ*_*g*_, and *d*_*g*_) and CCE had fair to relatively strong correlations with *K*_15_ and *K*_10_. It means that among the studied different soil physico-chemical attributes, the PSD-related parameters are more significant for predicting *K*_15_ and *K*_10_. Alternatively, by closing to saturation conditions, the soil structure-related parameters exhibited stronger correlations with *K*_*ψ*_, in which *K*_5_ and *K*_0_ were significantly related to SOM, pH, CCE, BD, and ln(SAR). Therefore, it can be concluded that with increasing tension (i.e., decreasing matric potential), the variation of unsaturated hydraulic conductivity can be better described by soil PSD-related parameters compared to the soil structure-related parameters. To the best of our knowledge, with decreasing tension from 15 to 0 cm the dependency of water flow to macropores increases. Therefore, soil structure strongly affects the *K*_*ψ*_ values. Simultaneously, the pores size distribution changes with soil structure [81]. In the well aggregated and structured soils, high amounts of macropores exist due to large aggregates. Therefore, it is expected that all factors that affect soil structure have a significant influence on *K*_0_ and to a lesser extent *K*_5_. As we respected, the *K*_5_ and *K*_0_ were more sensitive to variations of SOM, pH, BD, and ln(SAR). By increasing tension from 0 to 15 cm, the diameter of pores contributed in water flow, and consequently the effects of macropores on water flow decreased. At high tensions (like 10 and 15 cm in the present study), the soil primary particles are placed next to each other within aggregates, and different mesopores and micropores are formed. Therefore, dependencies of *K*_10_ and *K*_15_ to the PSD-related parameters were remarkably increased.

Tajik et al. [82] confirmed that soil particles can be dispersed and soil structure can be destroyed in response to high amounts of Na^+^ (SAR values), due to large hydrated radius. In addition, lower BD values might indicate more macropores in soils due to lower compaction. According to Vaezi et al. [83], Ostovari et al. [84,85], and Mozaffari et al. [86], the SOM and Ca^2+^ (represented by a high CCE) function as agents that bind mineral colloids together in order to facilitate flocculation. This process leads to the formation and stability improvement of aggregates. In accordance with our results, Kotlar et al. [13], in different Danish soil types, observed that log(*K*_10_) was powerfully related to PSD components (i.e., clay, sand, and silt contents) and the effects of these PSD parameters on log(*K*_0_) decreased, while the impact of BD (structure-related parameter) increased. A significant correlation between clay and SOM contents and the values of ln(*K*_0_) and ln(*K*_1_) was found by Yang et al. [87]. Further, they reported ln(*K*_5_) and ln(*K*_10_) significantly related to MWD. Santra et al. [88] reported the ln(*K*_0_) was significantly correlated with sand and clay contents, and BD in soils of Orissa, India.

Generally, the R values help us to clearly understand how each physico-chemical attribute is related to *K*_*ψ*_. In addition, the appeared parameters in the developed SMLR-PTFs for predicting *K*_*ψ*_ at each applied tension are justified by R values. It should be noted that some physico-chemical attributes have collinearity, and therefore the more effective ones would appear in the SMLR-PTFs for predicting *K*_*ψ*_.

### Predicting *K*_*ψ*_ by SMLR method

The *t*-test analysis revealed that there were no statistically significant differences between the mean values of validation and calibration *K*_*ψ*_ across all applied tensions. In addition, the distributions of *K*_*ψ*_ values at all applied tensions in the mentioned subsets were relatively similar. Considering these conditions, we ensure that the data included in the validation subset are not concentrated in a specific small range of all data. Therefore, the validation subset can surely examine the developed models by the calibration subset. The developed PTFs using studied physico-chemical attributes by applying SMLR approach for predicting *K*_*ψ*_ are provided in Table 4. It is worth mentioning that, for predicting *K*_*ψ*_, all studied soil physico-chemical attributes were imported to SMLR models as independent variables. The developed PTF for predicting *K*_15_ (Eq 20) using *d*_*g*_, *σ*_*g*_, silt, and CCE had R^2^_val_ (subscript val shows validation subset), NRMSE_val_ (%), NS_val_, and RPD_val_ values of 0.55, 27.4, 0.52, and 1.47, respectively. In addition to these mentioned easily measurable soil attributes, the ln(SAR) was appeared to PTF developed for predicting *K*_10_ (Eq 21). The *K*_10_-PTF predicted this property by R^2^_val_, NRMSE_val_ (%), NS_val_, and RPD_val_ values of 0.52, 25.9, 0.47, and 1.40, respectively. According to literature [89,90], the PSD and its related parameters can directly affect *K*_*ψ*_ at all tensions. For predicting *K*_5_ and *K*_0_, the silt, *σ*_*g*_, SOM, and CCE were appeared with positive sign, while the ln(SAR) was introduced with a negative sign in the PTFs developed (Eqs 22 and 23). Furthermore, to predict *K*_0_, the BD and pH contents were the influential factors by negative sign. By closing to saturated conditions, the macropores dependency of hydraulic conductivity increases [81]. Although the ln(Na^+^) showed a significant correlation with both *K*_5_ and *K*_0_, the impact of ln(SAR) was more pronounced (Fig 3). The R^2^_val_, NRMSE_val_ (%), NS_val_, and RPD_val_ values were 0.56, 18.1, 0.49, and 1.44 for *K*_5_ and 0.63, 25.4, 0.57, and 1.56 for *K*_0_ predictions, respectively.

**Table 4:** Developed PTFs for predicting *K*_*ψ*_ using easily measurable soil attributes by applying the SMLR method.

^†^ $K_{15}=-0.357+0.012(d_{g})+0.044(\sigma_{g})+0.003(\mathrm{Silt})+0.005(\mathrm{CCE})$	(20)
$K_{10}=-0.678+0.012(d_{g})+0.071(\sigma_{g})+0.008(\mathrm{Silt})+0.008(\mathrm{CCE})-0.068[\ln(\mathrm{SAR})]$	(21)
$K_{5}=-0.781+0.085(\sigma_{g})+0.012(\mathrm{Silt})+0.025(\mathrm{CCE})-0.17[\ln(\mathrm{SAR})]+0.24(\mathrm{SOM})$	(22)
$K_{0}=7.34+0.299(\sigma_{g})+0.035(\mathrm{Silt})+0.08(\mathrm{CCE})-0.632[\ln(\mathrm{SAR})]+1.08(\mathrm{SOM})-0.778(\mathrm{pH})-3.77(\mathrm{BD})$	(23)

†: The calculated performance criteria for PTFs developed to predict *K*_*ψ*_ were presented in Table 5 (the units of the presented parameters are the same as these mentioned in Table 3).

The PTFs (Eqs 20, 21 and 23) predicted *K*_15_, *K*_10_, and *K*_0_ by fair accuracy according to NRMSE (20–30%) and RPD (1.4–1.8) values. Furthermore, The *K*_5_-PTF (Eq 22) had good and fair accuracy based on NRMSE and RPD classifications, respectively. In addition, according to NS classification, the *K*_*ψ*_ across all of the applied tensions were predicted with unsatisfactory accuracy (NS_val_ < 0.65).

Overall, in comparison with *K*_15_, *K*_10_, and *K*_5_, the *K*_0_ was estimated more accurately. The moderate performance of *K*_*ψ*_-PTFs was anticipated due to the considerable variability of *K*_*ψ*_ even in soils with similar characteristics. This variability is greatly affected by the geometry of the pores, which contributes to the dependency of *K*_*ψ*_ to the mentioned factor [20]. Although we used some soil structure-related parameters in the present study, they are unable to fully capture the complexity of soil structure. Nevertheless, due to the challenging, time-consuming, and costly nature of measuring *K*_*ψ*_ [10], a model that provides even moderate prediction of this property can still be highly beneficial and valuable. In accordance with our results, in the western desert of Egypt, Gamie and De Smedt [12] discovered that the parameters related to soil structure (carbonate content, SAR, BD, and water content in saturated, field capacity, and wilting point conditions) exhibited stronger correlations with log(*K*_0_) as compared to the soil texture-related attributes (clay and silt contents). In contrast, Azadmard et al. [14] reported PTFs with R^2^ values of 0.17, 0.21, 0.14, 0.19, and 0.19 for prediction of *K*_15_, *K*_10_, *K*_5_, *K*_2_, and *K*_0_, respectively based on MLR approach using easily measurable soil attributes. Kotlar et al. [13] estimated log(*K*_0_) and log(*K*_10_) with R^2^ of 0.26 and 0.65, respectively using the SMLR method. In general, based on the statistical indices employed in this study, the *K*_*ψ*_ values at *ψ* of 15, 10, 5, and 0 cm were reasonably predicted using easily measurable soil attributes and applying SMLR method. The developed SMLR-PTFs can undergo testing, and subsequently be applied in other areas with the same soils.

### Predicting *K*_*ψ*_ by MLPNNs and RBFNNs methods

Table 5 summarized the performance of all applied approaches in the present study for predicting *K*_*ψ*_, i.e., SMLR, MLPNNs, and RBFNNs. As it was mentioned before, the imported independent variables to MLPNNs and RBFNNs models for predicting *K*_*ψ*_ at different applied tensions were these which appeared at Eqs (20) to (23). Regarding capability of MLPNNs approach to predict *K*_*ψ*_, the *K*_15_, *K*_10_, *K*_5_, and *K*_0_ were predicted by respectively R^2^_val_ values of 0.71, 0.78, 0.82, and 0.80. According to NRMSE classification, the *K*_15_, *K*_10_, and *K*_0_ were predicted fairly (20% < NRMSE_val_ < 30%) and *K*_5_ was predicted with good accuracy (NRMSE_val_ = 13.1%). The NS_val_ values showed acceptable accuracy (0.65 < NS_val_ < 0.80) for *K*_*ψ*_ at all applied tensions and RPD_val_ values showed very good predictions of *K*_5_ and *K*_0_ (2.05 and 2, respectively) and fair predictions of *K*_15_ and *K*_10_ (1.71 and 1.76, respectively).

**Table 5:** The performance criteria for predicting *K*_*ψ*_ using easily measurable soil attributes by applying the SMLR, MLPNNs, and RBFNNs approaches.

Model	Attribute	Calibration subset				Validation subset			
		R^2^	NRMSE	NS	RPD	R^2^	NRMSE	NS	RPD
SMLR-PTF	*K*_*15*_	0.58	25.4	0.58	1.56	0.55	27.4	0.52	1.47
	*K*_*10*_	0.53	24.9	0.52	1.45	0.52	25.9	0.47	1.40
	*K*_*5*_	0.60	16.4	0.60	1.60	0.56	18.1	0.49	1.44
	*K*_*0*_	0.67	24.2	0.67	1.75	0.63	25.4	0.57	1.56
MLPNNs	*K*_*15*_	0.78	22.3	0.68	1.79	0.71	23.7	0.65	1.71
	*K*_*10*_	0.81	18.3	0.70	1.85	0.78	23.6	0.66	1.76
	*K*_*5*_	0.84	11.7	0.81	2.33	0.82	13.1	0.75	2.05
	*K*_*0*_	0.82	18.4	0.80	2.26	0.80	21.3	0.74	2.00
RBFNNs	*K*_*15*_	0.67	22.8	0.63	1.63	0.58	24.7	0.59	1.50
	*K*_*10*_	0.78	19.6	0.70	1.74	0.62	24.3	0.48	1.62
	*K*_*5*_	0.80	16.0	0.78	2.07	0.78	17.1	0.74	1.87
	*K*_*0*_	0.80	19.8	0.76	2.05	0.77	22.5	0.73	1.63

On the other hand, The RBFNNs predicted *K*_15_, *K*_10_, *K*_5_, and *K*_0_ with R^2^_val_ values of 0.58, 0.62, 0.78, and 0.77, respectively. Similar to SMLR and MLPNNs approaches, the RBFNNs predicted *K*_5_ with good and the other studied *K*_*ψ*_s with fair accuracy based on NRMSE_val_ values. The NS_val_ values illustrated acceptable accuracy for *K*_5_ and *K*_0_ (NS_val_ values of 0.74 and 0.73, respectively) and unsatisfactory accuracy for *K*_15_ and *K*_10_ (NS_val_ < 0.65). Furthermore, based on RPD_val_ values, just *K*_5_ was predicted with good accuracy (RPD_val_ = 1.87) while, the *K*_15_, *K*_10_, and *K*_0_ were fairly predicted using mentioned ANNs approach.

Jian et al. [40] reported adjusted R^2^ of 0.53 and 0.33 related to the calibration and validation subsets, respectively to predict *K*_2_ by applying MLPNNs by 2 hidden layers (5 and 3 neurons for the first and second hidden layers) and using clay, silt, and sand contents as inputs. Merdun et al. [32] predicted *K*_0_ by R^2^ value of 0.52 using sand, silt, clay, BD, and pores with diameter > 30 μm, between 3–30 μm, and < 3 μm as input variables and cascade forward neural network. Ghanbarian-Alavijeh et al. [34] used one hidden layer MLPNNs to estimate *K*_0_ of UNSODA database. They found the mentioned approach successfully predicted *K*_0_ (with R^2^ values > 0.90) using clay and sand contents, BD, effective porosity and van Genuchten retention model parameters (θ_r_, *α*, and n) as input variables.

### Comparing potentials of SMLR, MLPNNs, and RBFNNs approaches to predict *K*_*ψ*_

**Fig 4:**
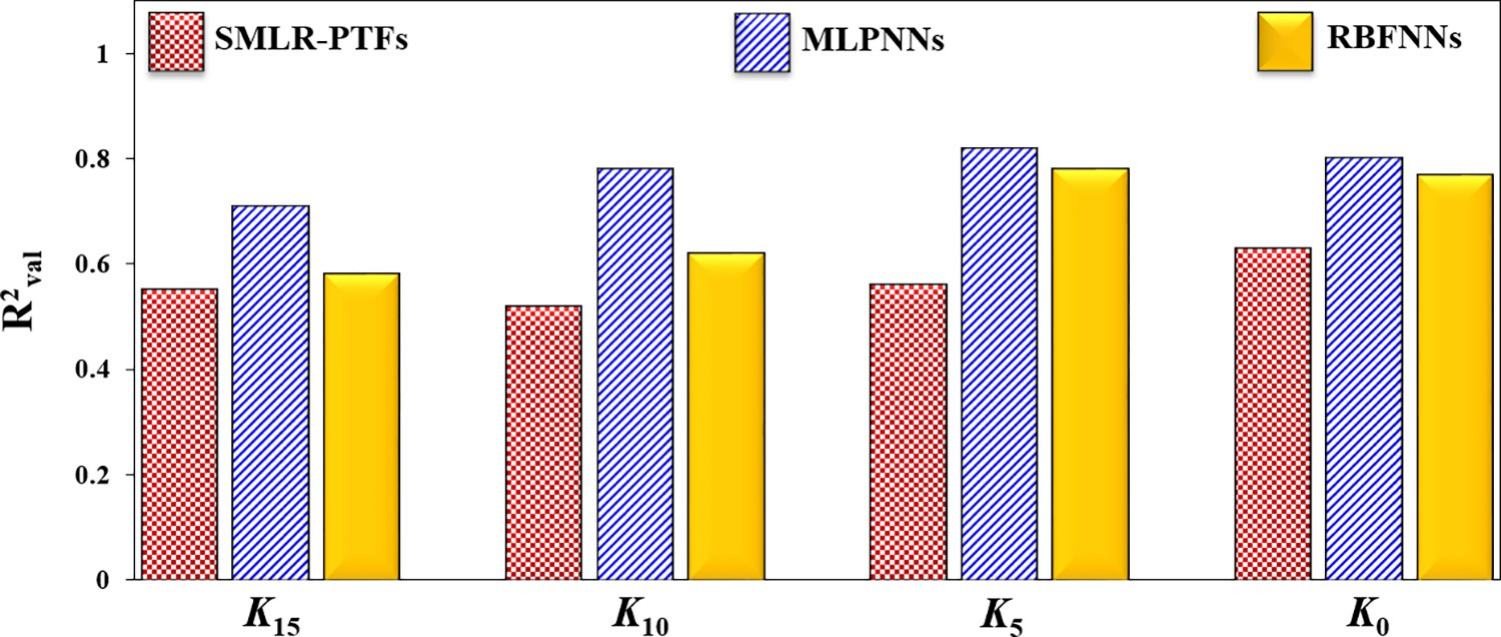
The column diagram of validation subset R^2^ (R2val) values for predicting *K*_15_, *K*_10_, *K*_5_, *K*_0_ using easily measurable soil attributes by applying SMLR, MLPNNs, and RBFNNs approaches.

Discovering the most powerful modeling approach for predicting hardly measurable soil attributes is very crucial in soil modeling studies. To the best of our knowledge, soil management practices are closely related to hardly measurable soil attributes. The limitations and difficulties for direct measurement of *K*_*ψ*_ lead soil scientists to investigate different modeling procedures and discover the most accurate ones to predict this important attribute. Therefore, it is beneficial to compare the potential of classical regression methods (like SMLR) with artificial intelligence algorithms (like MLPNNs and RBFNNs) to predict *K*_*ψ*_ at different tensions. Table 5 shows that the values of R^2^, NS, and RPD of different applied approaches for predicting *K*_*ψ*_ across all *ψ* were ranked as MLPNNs > RBFNNs > SMLR-PTFs and values of NRMSE was ranked as MLPNNs < RBFNNs < SMLR-PTFs. In order to better comparison between the capabilities of different approaches to predict *K*_*ψ*_, we provided the values of R^2^_val_ in Fig 4. Although the SMLR method provided simple and easy to use PTFs to predict *K*_*ψ*_ by using easily measurable soil attributes, their application may be limited due to relatively low accuracy. Of course, it should be pointed out that, for a property like hydraulic conductivity, these SMLR-PTFs are valuable due to difficulties in measuring *K*_*ψ*_ and its high variability. While, the MLPNNs approach showed a high capability (R^2^_val_ > 0.70) for prediction of *K*_*ψ*_ across all *ψ*. This could be attributed to the presence of non-linear relations among the soil hydraulic and physico-chemical attributes [14].

**Fig 5:**
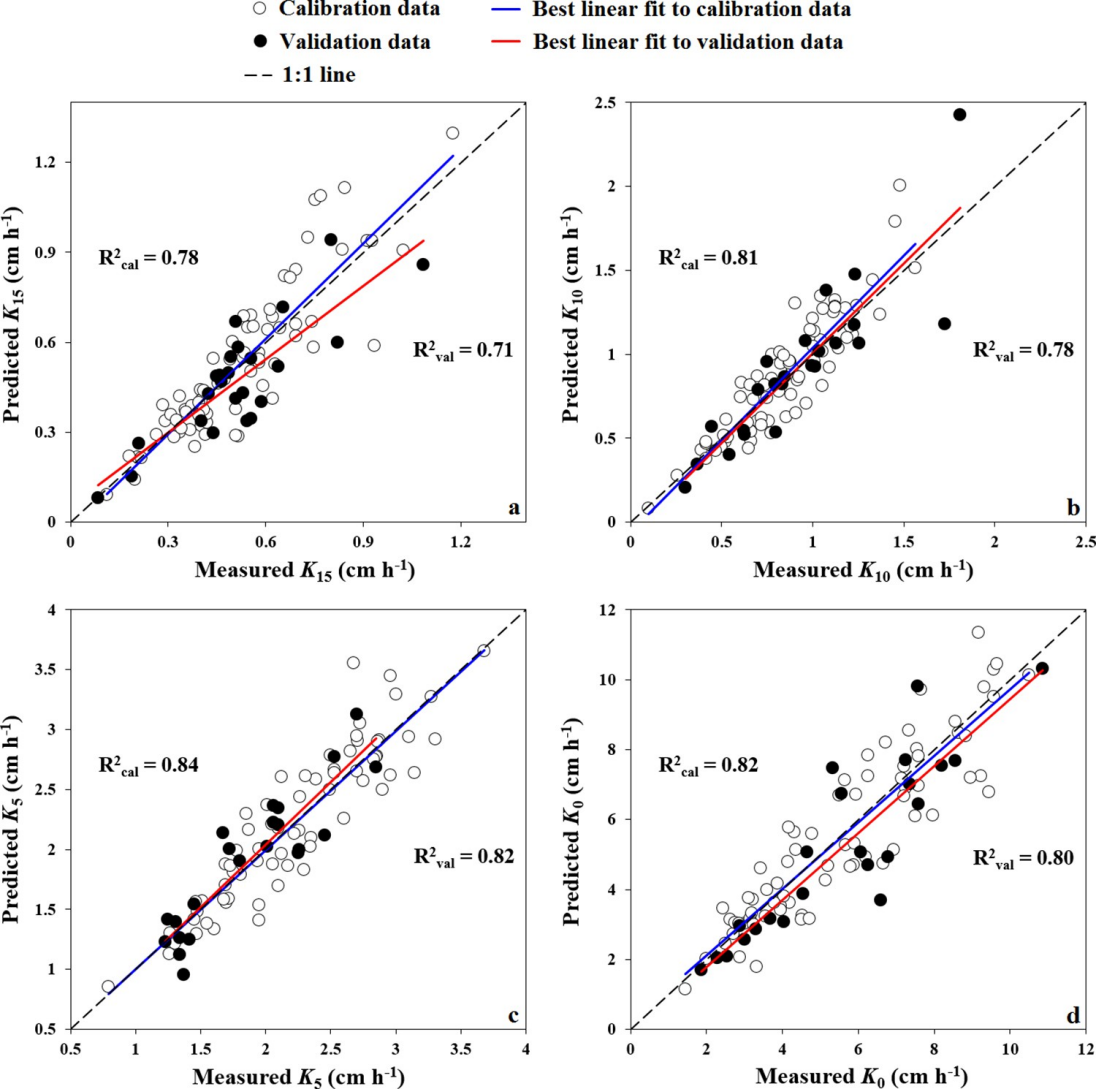
Scatter plots of the measured (observed) versus the predicted *K*_15_, *K*_10_, *K*_5_, *K*_0_ by applying MLPNNs approach as the best predictor and using easily measurable soil attributes.

Fig 5 shows the scatter plots of predicted against the observed (measured) *K*_*ψ*_ values across all *ψ* using MLPNNs as the best predictor approach. As can be seen, the points are near to 1:1 line for both validation and calibration subsets. In accordance with our results, Moosavi et al. [24] in soils of Fars Province, Iran, stated the capability of different approaches to predict sorptivity coefficient could be ranked as MLPNNs > RBFNNs > MLR regarding their accuracies and computational times. Furthermore, Shams Emamzadeh et al. [91] in soils of Tehran Province, Iran, found MLPNNs predicted *K*_0_ was more accurate than that of RBFNNs. While, Rezaei Arshad et al. [36], reported more capability of RBFNNs to predict *K*_0_ compared to MLPNNs and MLR methods in soils of Khuzestan Province, Iran. They predicted *K*_0_ by R^2^_val_ values of 0.68, 0.66, and 0.50 for RBFNNs, MLPNNs, and MLR approaches, respectively. Overall, the present study suggests using ANNs-based methods, especially MLPNNs, to predict saturated and near-saturated *K*_*ψ*_. The proposed modeling approaches can be applied to predict hardly measurable soil attributes in different regions. Generally, the strengths of the present study were: i) obtaining relatively accurate to accurate predictions of saturated and near-saturated *K*_*ψ*_ in calcareous soils using MLPNNs, and ii) showing the high capability of artificial intelligence algorithms for modeling *K*_*ψ*_ compared to a classical regression approach (SMLR). However, other studies can be performed for testing other machine learning modeling procedures to predict soil-water-related parameters, especially *K*_*ψ*_, and compare their potential with artificial intelligence algorithms. Furthermore, the developed SMLR-PTFs, can practically be tested in the same regions and then be used to predict *K*_*ψ*_. For the ANNs-based approaches used, we suggest developing special MLPNNs and RBFNNs algorithms for predicting *K*_*ψ*_ by using easily measurable soil attributes in different areas.

## Conclusions

The importance of *K*_*ψ*_ in water and chemical movements in the soil as well as the difficulties and time-consuming nature of its’ measurement caused performing the present study aimed to investigate the potential of SMLR, MLPNNs, and RBFNNs approaches to predict *K*_*ψ*_ at 15, 10, 5, and 0 (saturated condition) cm tensions using easily measurable soil attributes in calcareous soils. According to Pearson’s correlation coefficients, the PSD-related parameters were more correlated with *K*_15_ and *K*_10_ compared to structure-related parameters. The opposite results were obtained for *K*_5_ and *K*_0_. The MLPNNs approach provided the best prediction of *K*_*ψ*_ at all applied tensions by 0.71 ≤ R^2^_val_ ≤ 0.82 using recognized easily measurable soil attributes by SMLR-PTFs. In addition, the RBFNNs predicted *K*_*ψ*_ at all applied tensions acceptably to accurately with 0.58 ≤ R^2^_val_ ≤ 0.78. Although the developed SMLR-PTFs provided simple equations with relatively acceptable accuracy (0.52 ≤ R^2^_val_ ≤ 0.63), the present study recommends ANNs-based approaches (specifically MLPNNs) to predict *K*_*ψ*_ at different tensions due to higher capability. Generally, the accuracy of *K*_*ψ*_ prediction at all applied tensions by using different approaches and easily measurable soil attributes was ranked as MLPNNs > RBFNNs > SMLR-PTFs. The *K*_*ψ*_ is a crucial factor in water resources management and in the field of soil science and it should be accurately determined in arid and semi-arid regions. The calcareous soils are normally placed in arid and semi-arid regions that face shortage of fresh water for agricultural activities. In addition, lime (calcium carbonate) can help aggregation, improve soil structure, and subsequently affect *K*_*ψ*_ in calcareous soils. The findings of the present study, especially the developed SMLR-PTFs, can practically be tested and then used in the other similar soils. Furthermore, the soils of different areas can benefit from the applied ANNs approaches to receive accurate predictions of *K*_*ψ*_. Overall, machine learning algorithms (like those used in the present study) are beneficial for soil science researchers and professionals to find accurate PTFs for predicting and mapping the hardly measurable soil attributes in order to using in precision agriculture. For future works, we recommend paying more attention to predict soil hydraulic attributes in arid and semi-arid areas all over the world for using in water resources management and modeling the water and material transport within the soil. In addition, future studies can be performed to evaluate the performances of different machine learning approaches, like wavelet-neural networks, support vector regression, random forest, decision tree, cubist, k-nearest neighbors, etc. for predicting *K*_*ψ*_. To conclude the present study, the readers should consider two important issues: i) accurate predictions of saturated and near-saturated hydraulic conductivity in arid and semi-arid regions, and ii) application of powerful machine learning algorithms for accurate prediction of hardly measurable soil attributes.

## Supporting information

S1 File Highlights and Raw data are available as supplementary files. (DOC)

S1 Raw data (XLS)

## Abbreviations

ANNs: Artificial neural networks
BD: Bulk density
Ca^2^-+: Water-soluble calcium
CCE: Calcium carbonate equivalent (lime)
CV: Coefficient of variations
D: Fractal dimension of particle-size distribution
d_g_: Geometric mean of particles size diameter
EC: Electrical conductivity of saturated extract
FFNNs: Feed-forward neural networks
K^+^: Water-soluble potassium
K_ψ_: Hydraulic conductivity at tension *ψ*
*K*_0_: Hydraulic conductivity at tension 0 cm (saturated hydraulic conductivity)
*K*_5_: Hydraulic conductivity at tension 5 cm
*K*_10_: Hydraulic conductivity at tension 10 cm
*K*_15_: Hydraulic conductivity at tension 15 cm
KR: Kurtosis coefficient
KS: Statistic of Kolmogorov-Smirnov normality test
Mg^2+^: Water-soluble magnesium
Max: Maximum
Min: Minimum
MLPNNs: Multilayer perceptron neural networks
MWD: Mean weight diameter of aggregates
n: Number of data
Na^+^: Water-soluble sodium
NRMSE: Normalized root mean square error
NS: Nash-Sutcliffe coefficient
pH: pH of saturated paste
PSD: Particle-size distribution
PTF: Pedotransfer function
Q_ψ_: Volume of water infiltrates into the soil per unit time at tension *ψ*
r: Radius of the disc infiltrometer apparatus
R: Pearson’s correlation coefficient
R^2^: Determination coefficient
RBFNNs: Radial-basis function neural networks
RPD: Residual prediction deviation
SAR: Sodium adsorption ratio
SK: Skewness coefficient
SMLR: Stepwise multiple linear regression
SOM: Soil organic matter
Subscript cal: Calibration subset
Subscript val: Validation subset
VC: Variability class according to Wilding’s classification
α: Sorptive number
σ_g_: Geometric standard deviation of particles size diameter
ψ: Applied tension
