# Supplementary material for: Predicting saturated and near-saturated hydraulic conductivity using artificial neural networks and multiple linear regression in calcareous soils
Source: PLoS One. 2024 Jan 10;19(1):e0296933. doi: 10.1371/journal.pone.0296933 (PMC10781043; doi:10.1371/journal.pone.0296933)
Supplement: S1 File — (DOC) [file pone.0296933.s001.doc]

**Highlights**

- Easily measurable attributes (EMA) were correlated with hydraulic conductivity (K_ψ_)
- K_ψ_ was acceptably predicted by stepwise multiple linear regression (SMLR) using EMA
- Multilayer perceptron neural networks (MLPNNs) accurately predicted K_ψ_ using EMA
- Radial-basis function NNs (RBFNNs) acceptably to accurately predicted K_ψ_ using EMA
- Accuracy of K_ψ_ prediction by different methods was ranked as: MLPNNs > RBFNNs > SMLR
